# Influence of Titanium Dioxide Nanoparticles on the Sulfate Attack upon Ordinary Portland Cement and Slag-Blended Mortars

**DOI:** 10.3390/ma11030356

**Published:** 2018-02-28

**Authors:** Atta-ur-Rehman, Abdul Qudoos, Hong Gi Kim, Jae-Suk Ryou

**Affiliations:** Department of Civil and Environmental Engineering, Hanyang University, 222, Wangsimni-ro, Seongdong-gu, Seoul 04763, Korea; attabrcian@gmail.com (A.R.); qudoos.engnr@gmail.com (A.Q.); dmkg1404@naver.com (H.G.K.)

**Keywords:** titanium dioxide, nanoparticles, photocatalysis, sulfate attack, mortar, cement, blast furnace slag, expansion, deterioration, microcracks

## Abstract

In this study, the effects of titanium dioxide (TiO_2_) nanoparticles on the sulfate attack resistance of ordinary Portland cement (OPC) and slag-blended mortars were investigated. OPC and slag-blended mortars (OPC:Slag = 50:50) were made with water to binder ratio of 0.4 and a binder to sand ratio of 1:3. TiO_2_ was added as an admixture as 0%, 3%, 6%, 9% and 12% of the binder weight. Mortar specimens were exposed to an accelerated sulfate attack environment. Expansion, changes in mass and surface microhardness were measured. Scanning Electron Microscopy (SEM), Energy Dispersive Spectroscopy (EDS), X-ray Diffraction (XRD), Thermogravimetry Analysis (TGA) and Differential Scanning Calorimetry (DSC) tests were conducted. The formation of ettringite and gypsum crystals after the sulfate attack were detected. Both these products had caused crystallization pressure in the microstructure of mortars and deteriorated the mortars. Our results show that the addition of nano-TiO_2_ accelerated expansion, variation in mass, loss of surface microhardness and widened cracks in OPC and slag-blended mortars. Nano-TiO_2_ containing slag-blended mortars were more resistant to sulfate attack than nano-TiO_2_ containing OPC mortars. Because nano-TiO_2_ reduced the size of coarse pores, so it increased crystallization pressure due to the formation of ettringite and gypsum thus led to more damage under sulfate attack.

## 1. Introduction

### 1.1. Background

Titanium dioxide (TiO_2_) is a white and inorganic material. It is used as an effective photocatalyst and can be activated by light radiation to degrade organic and inorganic pollutants present in the water and air through oxidation-reduction process. In recent decades, TiO_2_ has been added to concrete during mixing to yield a product with self-cleaning and air purifying properties [1]. TiO_2_ can clean the surface of concrete by degrading certain pollutants deposited on its surface in the presence of sunlight and can also convert harmful gases like nitrous oxides and volatile organic compounds (VOCs) to less harmful products [2,3]. Concrete with the addition of TiO_2_ has been referred to in the literature as self-cleaning, air purifying, or photocatalytic concrete. Photocatalytic concrete has been effectively used in the pavement blocks, tunnels, sidewalks and external walls of buildings for air purifying purposes and to maintain the aesthetic properties of buildings [4,5]. Additionally, ground granulated blast furnace slag (GGBFS) is an industrial byproduct with a white color. Use of slag in cementitious materials is environmentally beneficial as it helps in reducing carbon dioxide emissions and energy use due to cement production [6]. It can be used in concentrations up to 30% to replace white Portland cement for architectural effects in white concrete structures [7]. The aesthetic appeal of such structures can be preserved by adding nano-TiO_2_ [4,8]. Simultaneous use of TiO_2_ and slag in cementitious materials can inherit them with beneficial characteristics of both slag and TiO_2_.

Sulfate attack is one of the most aggressive environmental conditions to address for concrete. Sulfate ions present in the soil, run-off water, seawater, groundwater and sewer lines can move to the interior of the concrete through pores and react with unhydrated and hydrated alumina phases, portlandite (CH), calcium silicate hydrate (CSH) to produce ettringite and gypsum. Incoming SO_4_^2−^ ions react with calcium alumino monosulfate phases and produce ettringite (3CaO·Al_2_O_3_·3CaSO_4_·32H_2_O) according to Equation (1) [9]:

SO_4_^2−^ + 4CaO·Al_2_O_3_·SO_3_·12H_2_O + 2Ca^2+^ + 20H_2_O → 3CaO·Al_2_O_3_·3CaSO_4_·32H_2_O (1)

Sodium sulfate reacts with portlandite to produce gypsum and sodium hydroxide according to Equation (2):

Na_2_SO_4_ + Ca(OH)_2_ → CaSO_4_·2H_2_O + NaOH (2)

Gypsum reacts with hydrated products such as calcium aluminates, calcium sulfoaluminate (monosulfate- C_4_ASH_12–18_) or tricalcium aluminate, (unhydrated phase in cement clinker) to produce ettringite, as given in the Equations (3)–(5):

4CaO·Al_2_O_3_·13H_2_O + 3(CaSO_4_·2H_2_O) + 13H_2_O → 3CaO·Al_2_O_3_·3CaSO_4_·32H_2_O + Ca(OH)_2_ (3)

4CaO·Al_2_O_3_·SO_3_·12H_2_O + 2(CaSO_4_.2H_2_O) + (10 − 16) H_2_O → 3CaO·Al_2_O_3_·3CaSO_4_·32H_2_O (4)

3CaO·Al_2_O_3_ + 3(CaSO_4_·2H_2_O) + 26H_2_O → 3CaO·Al_2_O_3_·3CaSO_4_·32H_2_O (5)

Ettringite is an expansive product and causes expansion in the hardened cement paste. Expansion due to gypsum is disputed [10], researchers agree that gypsum softens mortar and reduces its strength [11]. If the expansive stresses from the formation of new products exceed the tensile strength of the hardened concrete, then microcracks are formed. These microcracks allow greater transport of sulfate ions from the external environment to the interior of the concrete and accelerate the sulfate attack. Sulfate attack increases porosity, reduces strength, softens concrete, changes mass, causes expansion, cracking and spalling. The severity and nature of these defects are dependent on factors such as cement type and composition, water/binder ratio, presence and amount of supplementary cementitious materials, porosity, permeability, ambient temperature, the concentration of sulfate ions and types of cations, such as sodium or magnesium. More details relating to the mechanism of sulfate attack can be found in the previous reviews [9,11,12,13].

### 1.2. Research Significance

During service life, photocatalytic concrete is in contact with soil or water; as both soil and water are sources of sulfate attack, there is a high possibility of such an attack. The addition of nano-TiO_2_ alters the mechanical properties and the microstructure of the concrete [14]; therefore, it is necessary to investigate sulfate attack on nano-TiO_2_ containing concrete to design efficient photocatalytic structures and avoid premature failure. Research into sulfate attack on photocatalytic mortars is sparse. In this study, we investigate the effects of nano-TiO_2_ on the sulfate attack resistance of pure Portland cement mortars and slag-blended mortars at two exposure temperatures. Nano-TiO_2_ was added to the mortars at binder weight percentages of 0%, 3%, 6%, 9% and 12%. Other researchers have also used large TiO_2_ dosage in their studies [14,15,16,17,18]. Lower dosage of TiO_2_ in the cementitious materials may be insufficient to ensure self-cleaning and air purification at longer ages. Therefore, higher dosage of TiO_2_ was chosen. The mortars were immersed in 10% sodium sulfate solution at 5 and 25 °C and expansion, changes in mass and loss of surface microhardness were measured. SEM, EDS, XRD, TGA and DSC tests were conducted on deteriorated specimens. 

## 2. Experimental Program

### 2.1. Materials and Mix Proportion

Titanium dioxide used in this study was produced by Cristal France SAS (Thann, France). According to Cristal Corporate, the content of anatase phase in the product was more than 99%, the size of nanoparticles ranged from 10~60 nm and specific surface area of the powder was 85.6 m^2^/g. Ordinary Portland cement and ground granulated blast furnace slag were purchased from a local company (Dongyong Cement, Co., Ltd., Seoul, Korea). The chemical compositions of the cement and slag are given in Table 1. 

Control and binder/TiO_2_ composite mortars were made by adding TiO_2_ as 3%, 6%, 9% and 12% of the binder weight. Water/binder ratio was fixed to 0.4, a polycarboxylate ether-based water reducing admixture (0–1.2% of the binder weight) was added to adjust the fluidity of the mortars. Mix proportions are shown in Table 2. The TiO_2_ particles were first deagglomerated and dispersed in water through ultrasonication using a sonic probe for at least 45 min. Then water reducing admixture was added in the water and stirred for one more minute. This mixture was slowly added to the sand and binder in the mortar mixer and then mortar specimens were formed. 

### 2.2. Test Procedure and Specimen Preparation

**Figure 1:**
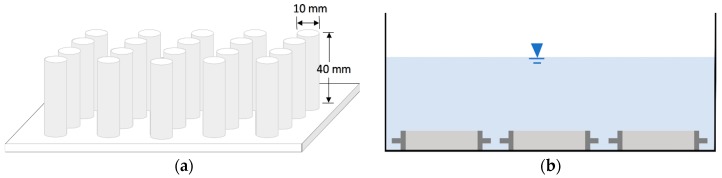
(**a**) A schematic diagram of molds prepared in the laboratory for casting cylindrical mortar specimens; (**b**) Mortar specimens immersed in Na_2_SO_4_ solution.

ASTM C 1012 [19] is one of the test methods used to evaluate sulfate resistance. According to this method, mortar prisms with dimensions of 25.4 mm × 25.4 mm × 279.4 mm with pins at both ends are prepared and stored in limewater until reaching a strength of 20 MPa. They are then stored in 5% sodium sulfate (Na_2_SO_4_) solution and any expansion is measured. The ASTM C 1012 process takes several months to a year to complete; many accelerated tests have been proposed using small specimen sizes and higher concentrations of sulfate solution. A summary of these tests can be found in the work of Kim Van Tittelboom et al. [20]. Ferraris et al. [21] developed a new accelerated test technique for measuring the sulfate resistance of mortar. Here, authors used their test method with some modifications. They had immersed 10 mm × 10 mm × 40 mm rectangular prism specimens in 5% Na_2_SO_4_ solution. In this study, cylindrical mortar specimens with a diameter of 10 mm and length 40 mm were immersed in 10% Na_2_SO_4_ solution. Because expansion depends on size rather than shape, specimen size is a crucial factor in evaluating sulfate resistance [20,22], although crack patterns might differ in cylindrical and rectangular prisms. To cast the specimens, molds were made in the laboratory using the barrel of syringes with an internal diameter of 10 mm. The barrel of a syringe was cut from the plunger and needle side to a length of 40 mm and was then fixed on a glass plate with epoxy, as shown in Figure 1a. The mortar was cast in molds in four layers and each layer was compacted 20 times with a 2 mm steel rod and the surface was leveled. Twenty specimens were made for each mix proportion, for a total of 200 specimens. Mortar cubes and prisms were casted to measure compressive and flexural strengths After 48 h of casting, the mortar specimens were removed from the molds and placed in saturated limewater for 28 days. Four specimens of each mix proportion were left immersed in saturated limewater while other specimens were selected for immersion in Na_2_SO_4_ solution. The studs were firmly fixed on the ends of these mortar specimens with epoxy. To allow sulfate ions to attack only from the sides, the epoxy was applied to both end faces of the specimens. Then they were immersed in 10% Na_2_SO_4_ solution as shown in Figure 1b, which was renewed every other week. The volumetric ratio of the solution to the test specimens was 20:1. Half of the specimens were stored at a temperature of 5 ± 1 °C and a half at 25 ± 1 °C. Both temperatures were maintained constant for the entirety of the study period. Compressive and flexural strength tests were conducted according to ASTM C109 [23] standard and ASTM C348 [24], respectively, at the age of 28 days. Water absorption was measured at the age of 28 days [25].

### 2.3. Expansion and Mass Variation

The length and mass of the specimens were measured every seventh day during the immersion period using Equations (6) and (7):

Increase in length at (t) = (L_t_ − L_i_)/L_i_ × 100 (6)

Increase in mass at (t) = (M_t_ − M_i_)/M_i_ × 100 (7)

where L is the length, t is the time, i is the initial and M is the mass. The test was stopped after 84 days of immersion. 

### 2.4. Vickers Microhardness 

**Figure 2:**
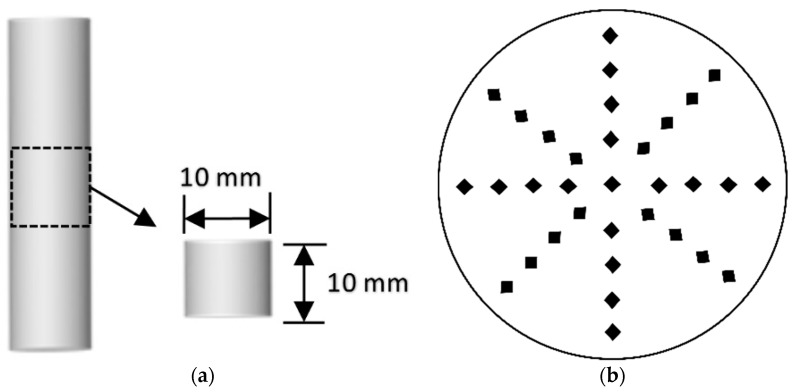
(**a**) The preparation of the sample used for microhardness; (**b**) Cross-sectional schematics of the sample used in the Vickers microhardness test.

The Vickers microhardness test is a common surface hardness characterization technique [26]. In this method, a static load is applied for a fixed time on the surface of the material using an indenter and the area of indentation is calculated. The Vickers hardness indenter resembles a diamond pyramid in shape. The Vickers hardness value is the ratio of applied load to indentation contact area and is calculated according to Equation (8):

HV = 2Psin(ɸ2)/D^2^ = 1.854P/D^2^, (8)

where HV is the Vickers hardness value, P is the load (kgf), D is the mean diagonal of the indentation pyramid (mm) and ɸ = 136°. To measure the Vickers microhardness, samples with a diameter of 10 mm and depth of 10 mm were obtained by cutting cylindrical specimens with a diamond saw cutter as shown in Figure 2a. Both ends of the obtained samples were successively polished with 400, 800, 1200 and 1500 grit polishing papers and placed in an oven at 55 °C for 24 h to remove the humidity. A load of 0.1 kgf was applied and maintained for 10 s. Microhardness values were measured at 1, 2, 3, 4 and 5 mm from the external surface, as shown in Figure 2b. A minimum of 8 readings were taken at each depth. The test was conducted using a Vickers hardness tester THV-1MD (Capital Instrument, Beijing, China). 

### 2.5. Microscopic Observation and Damage Rating

Cracks were observed with a microscope, EGVM-452 M (EG Tech, Seoul, Korea). The samples were observed every four weeks both with the unaided eye and through the microscope to quantify the damage. The specimens were rated on a scale from 0 to 7, as given in Table 3.

### 2.6. XRD, SEM, EDS, TGA and DSC Analyses

The damaged specimens were analyzed using X-ray diffraction (XRD, Rigaku, Tokyo, Japan), Scanning Electron Microscopy (SEM; Model S-3000 N, Hitachi, Tokyo, Japan) equipped with Energy Dispersive Spectroscopy (EDS) tester after 84 days of immersion. For XRD analysis, controlled and deteriorated mortar samples were ground, passed through a 200-µm sieve. The XRD test was conducted using the RINT D/max 2500 X-ray diffractometer at a voltage of 40 kV, current of 30 mA and a scanning speed of 2°/min using CuKα X-rays with a wavelength of 1.54 Å. For SEM and EDX analyses, samples were prepared according to Sarkar et al.’s work [27]. TGA and DSC analysis were conducted on the powdered samples obtained from sliced samples as shown in Figure 2a. The instrument used was Thermogravimetric Analyzer and Differential Scanning Calorimeter, DSC SDT Q600 (TA Instruments, New Castle, DE, USA). Samples were heated from room temperature to 1000 °C at the rate of 10 °C/min, nitrogen was purged at a flow rate of 100 mL/min. Powdered samples were prepared according to Lothenbach et al.’s work [28].

## 3. Results and Discussion

### 3.1. Compressive, Flexural Strengths and Water Absorption

**Figure 3:**
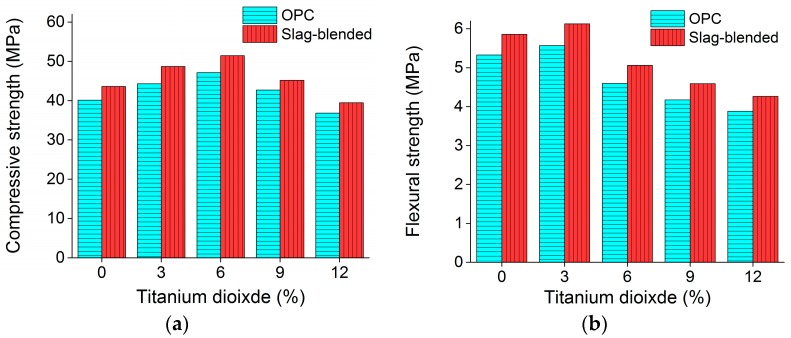
Variation of mechanical properties with the addition of nano-TiO_2_ in mortars at the age of 28 days (**a**) compressive strength (**b**) flexural strength.

**Figure 4:**
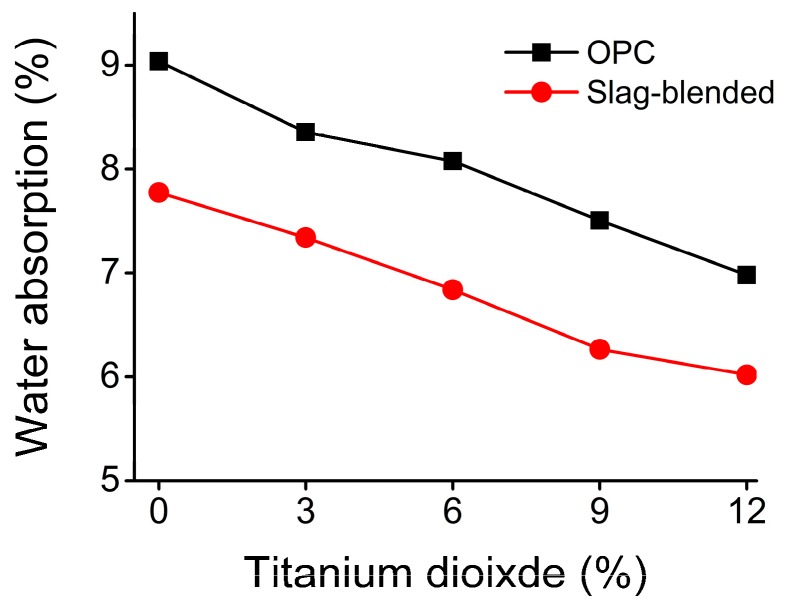
Variation of water absorption with the addition of nan-TiO_2_ in the mortars.

Figure 3 shows the variation of compressive and flexural strengths with the addition of nano-TiO_2_ in OPC and slag-blended mortars. Adding more than 6% nan-TiO_2_ as weight of the binder led to a reduction in compressive strength of mortars while adding more than 3% nan-TiO_2_ as weight of the binder led to a reduction in flexural strength of mortars. Increase in compressive strength was due to filler effect of nano-TiO_2_ [29]. The water absorption results are shown in Figure 4 which shows that water absorption is reduced with the addition of nano-TiO_2_ in both type of mortars. This positive effect can be ascribed to the nano-TiO_2_ particles, which acted as fillers, served as nucleation sites for the hydration reaction, hydration products precipitated around them and disconnected pores in the paste. The reduction in water absorption due to slag addition can be ascribed to the their later hydration and formation of secondary CSH in capillary pores [30]. 

### 3.2. Expansion and Mass Variation

**Figure 5:**
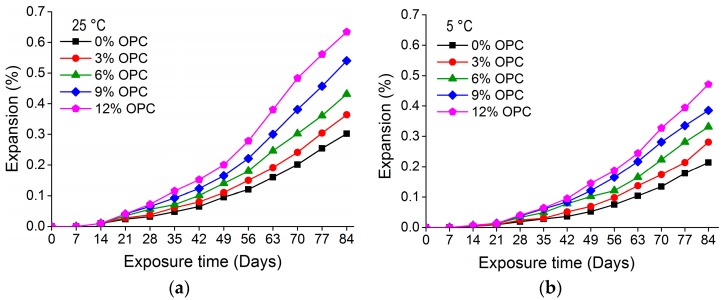
Expansion in nano-TiO_2_ containing OPC mortars immersed in 10% Na_2_SO_4_ solution at (**a**) 25 °C (**b**) 5 °C.

**Figure 6:**
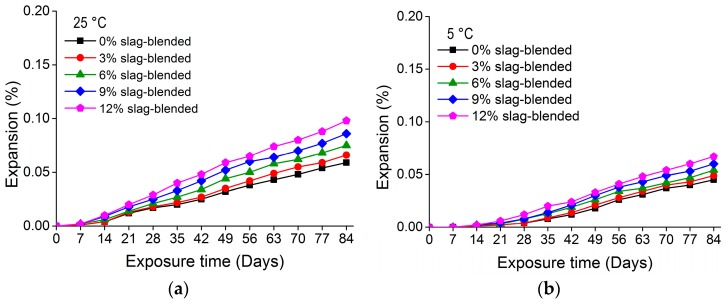
Expansion in nano-TiO_2_ containing slag-blended mortars immersed in 10% Na_2_SO_4_ solution at (**a**) 25 °C (**b**) 5 °C.

Figure 5 and Figure 6 show the expansion in nano-TiO_2_ containing OPC and slag-blended mortars immersed in Na_2_SO_4_ solution at 25 °C and 5 °C for 84 days, respectively. The results show an increase in expansion rate with the addition of nano-TiO_2_. At 25 °C, the expansion in 0% OPC mortars was 0.30%, while the expansion in 3%, 6% and 9% OPC mortars was 0.36%, 0.43% and 0.54%, respectively, after 84 days. The increase in expansion with the increase of nano-TiO_2_ occurred at both temperatures (25 and 5 °C). The comparison of expansion in OPC and slag-blended mortars showed that nano-TiO_2_ containing OPC mortars expanded more than nano-TiO_2_ slag-blended mortars. The expansion pattern differed between nano-TiO_2_ slag-blended and nano-TiO_2_ containing OPC mortars; whereas former exhibited a uniform rate of expansion but OPC mortars showed slow expansion initially and faster expansion at later times. Slag improves performance by acting as a physical filler and pozzolanic material [31]. Therefore nano-TiO_2_ containing slag mortars were more resistant to expansion. Storage temperature also showed a prominent effect on the rate of expansion of TiO_2_ containing mortars. 

**Figure 7:**
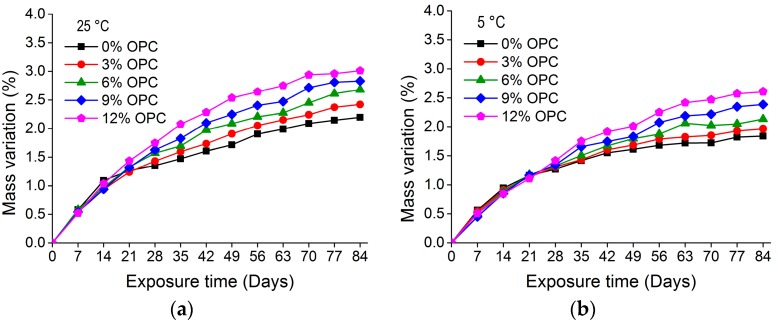
Mass variation in nano-TiO_2_ containing OPC mortars immersed in 10% Na_2_SO_4_ solution at (**a**) 25 °; (**b**) 5 °C.

**Figure 8:**
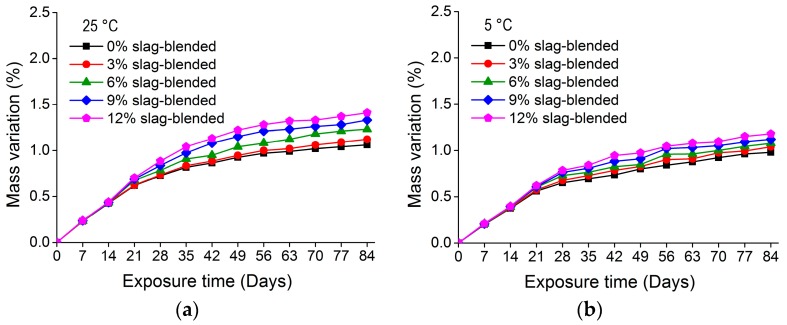
Mass variation in nano-TiO_2_ containing slag-blended mortars immersed in 10% Na_2_SO_4_ solution at (**a**) 25 °C; (**b**) 5 °C.

Figure 7 and Figure 8 show the mass variation for nano-TiO_2_ containing OPC and slag-blended mortars immersed in Na_2_SO_4_ solution at 25 and 5 °C, respectively. The addition of TiO_2_ in mortars resulted in an additional mass gain. After immersion for 84 days at 25 °C, 12% OPC and 12% slag-blended mortars gained 3.01% and 1.41% mass, respectively, while 0% OPC and 0% slag-blended mortars gained 2.19% and 1.06% mass, respectively. The increase in mass of mortars may be due to absorption of sodium sulfate solution and precipitation of gypsum and ettringite in pores and cracks. The effects of TiO_2_ addition on the rate of mass increase were negligible in first few weeks but prominent at later stages. The increase in mass was higher in mortars immersed in sulfate solution at higher temperatures. The addition of slag refined pores and reduced capillary pores and their connectivity [6,32,33], hence reducing the absorption of sodium sulfate solution. Therefore, nano-TiO_2_ containing slag-blended mortars were less vulnerable to the formation of gypsum and ettringite and exhibited less expansion and variation in mass.

### 3.3. Microhardness

**Figure 9:**
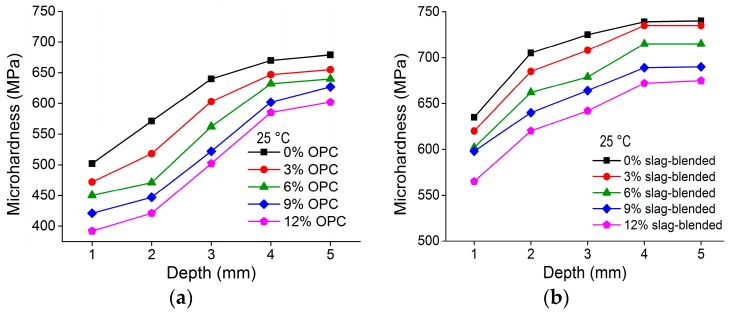
Variation in microhardness along depth in (**a**) nano-TiO_2_ containing OPC mortars and (**b**) nano-TiO_2_ containing slag-blended mortars immersed in 10% Na_2_SO_4_ solution at 25 °C.

The variation in Vickers hardness in OPC and slag-blended mortars immersed in 10% Na_2_SO_4_ solution at 25 °C is shown in Figure 9. It shows a greater loss of surface hardness with the addition of nano-TiO_2_. This figure also shows that sulfate attack caused a greater loss of surface hardness in nano-TiO_2_ containing OPC mortars than in nano-TiO_2_ containing slag-blended mortars. In OPC mortars, most of the depth is affected and the peripheral area was softened with respect to the inner depth, while in slag-blended mortars, the loss of surface hardness value extended up to 3 mm depth. This indicates the resistance of nano-TiO_2_ containing slag-blended mortars to sulfate attack and an increase in sulfate damage due to the addition of nanoparticles.

### 3.4. Microcracks 

**Figure 10:**
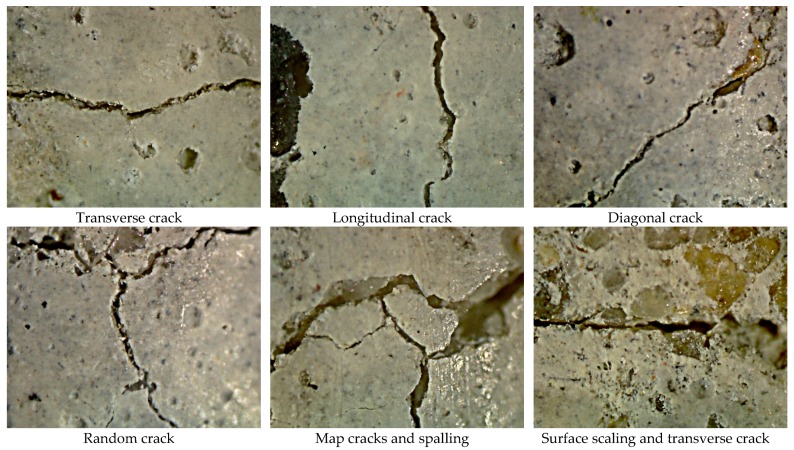
A representative view of cracks formed on the surface of 12% OPC mortars observed under a light microscope after 84 days of immersion in Na_2_SO_4_ solution at 25 °C.

**Figure 11:**
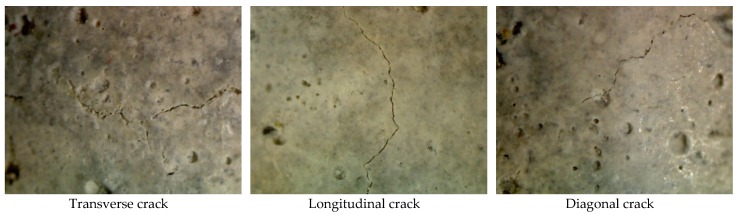

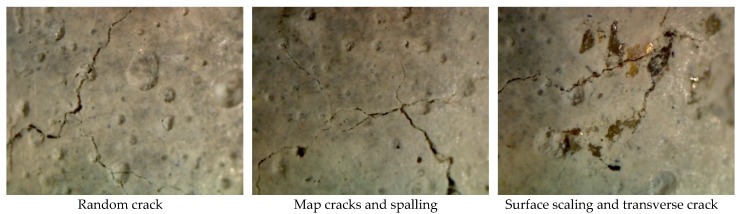
A representative view of cracks formed on the surface of 12% slag-blended mortars observed under a light microscope after 84 days of immersion in Na_2_SO_4_ solution at 25 °C.

Various cracks were formed on the surface of the specimens with respect to the axis of specimens, including perpendicular, diagonal and parallel. These cracks were referred to as transverse, diagonal and longitudinal, respectively. Cracks that changed direction from transverse to diagonal or longitudinal were identified as random cracks. Map cracks, which run in different directions and form hexagonal and irregular patterns on the surface of mortar, were also formed. At some points, spalling had occurred in the form of pop-outs and conical depressions due to the surrounding of an aggregate, or layer of the mortar, by a map crack. A representative view of the surface cracks formed on the specimens is shown in Figure 10 and Figure 11, all of which occurred as a result of sulfate attack. Due to sulfate attack, transverse and diagonal cracks were numerous and wider than other types of cracks, followed in severity by map cracks and longitudinal cracks. This suggests that expansion of mortar samples is the dominant damage caused by sulfate attack. Table 3 shows the damage rating of nano-TiO_2_ containing mortars on a scale from 0 to 7. The details of scale can be seen in the footnote of table. Severity of cracks varied among mix proportions. For example, 12% and 9% OPC mortars were severly damaged after 84 days compared to 3% and 0% OPC mortars. The same pattern was found in slag-blended mortars. 

### 3.5. XRD, SEM, EDS, TGA and DSC Analyses

**Figure 12:**
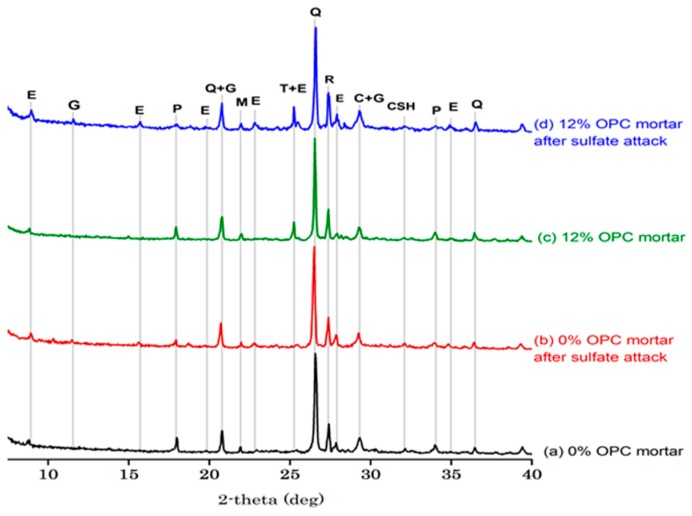
XRD pattern of OPC mortars (E = ettringite, G = gypsum, P = portlandite, Q = quartz, M = monosulfate, T = TiO_2_ (anatase), R = rutile, C = calcite, CSH = calcium-silicate-hydrate).

**Figure 13:**
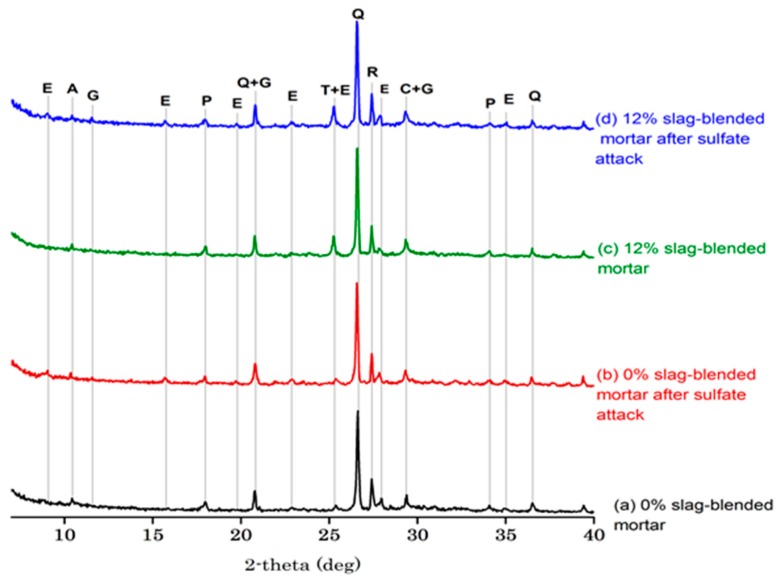
XRD pattern of slag-blended mortars (E = ettringite, A = tetra calcium aluminate hydrate, G = gypsum, P = portlandite, Q = quartz, T = TiO_2_ (anatase), R = rutile, C = calcite, CSH = calcium-silicate-hydrate).

Figure 12 and Figure 13 show XRD graphs of the 0% and 12% OPC mortar and 0% and 12% slag-blended mortars stored in saturated limewater and Na_2_SO_4_ solution after 84 days of immersion at 25 °C, respectively. Ettringite, portlandite, quartz, calcite, anatase (TiO_2_), rutile, monosulfate and hydrated tetra calcium aluminate were observed in XRD graphs of 0% and 12% OPC and slag-blended mortars before immersion, as shown in Figure 12a,c and Figure 13a,c. Ettringite is a hydration product formed due to the reaction of gypsum present in cement clinker and forms when concrete is in a plastic state. Its formation provides dimensional stability to the mortar, as it compensates for chemical shrinkage. However, its formation in later stages in hardened concrete is detrimental [34]. The peaks of portlandite in the XRD pattern of slag-blended mortars (Figure 13a,c) were lower than OPC mortars (Figure 12a,c) due to the reaction of slag with portlandite, which consumed them during a pozzolanic reaction. XRD patterns of damaged OPC (Figure 12b,d) and slag-blended mortars (Figure 13b,d) showed new peaks of ettringite and gypsum after immersion in sulfate solution, existing peaks of ettringite were increased and portlandite peaks were decreased in intensity. The appearance of new peaks of ettringite and gypsum confirmed that expansion and cracking were due to the formation of ettringite and gypsum. Although slag-blended mortars had more Al_2_O_3_ content than OPC mortars but they showed resistance to sulfate attack than OPC mortars. In the case of hydration of slag, Al^3+^ is bound in calcium silicate gel as C-A-S-H which shelters it form attack of incoming sulfate ions [35]. Furthermore, less monosulfate is produced in slag mortars compared to OPC mortars [13]. Monosulfate serves as an important source of Al^3+^ ions for the formation of ettringite after attack of incoming sulfate ions [13]. Therefore, less ettringite is produced in slag blended mortars.

**Figure 14:**
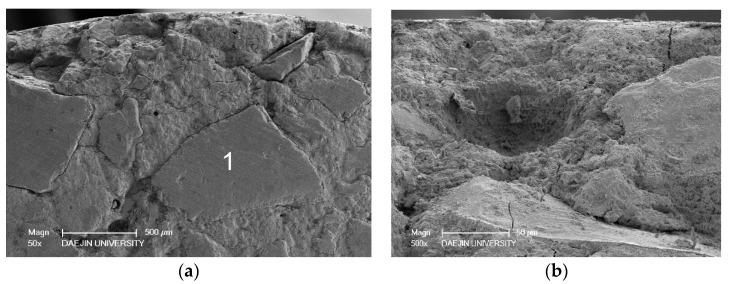
SEM images of 12% OPC mortar after sulfate attack (**a**) Cracks are more visible in the peripheral zone compared to the inner zone, visible crack on the outer side of aggregate 1 compared to its inner side. (**b**) A crack originating from the outer surface moving toward the inner core.

**Figure 15:**
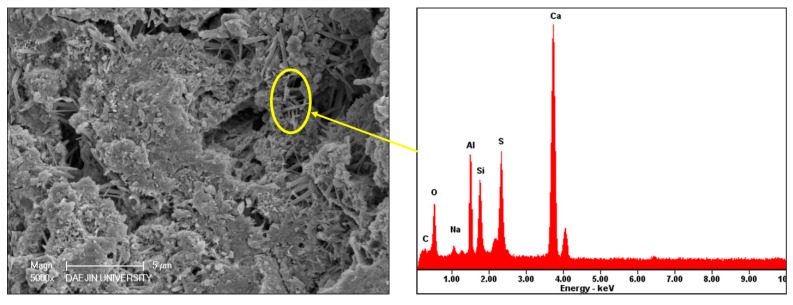
SEM image and EDS analyses of ettringite crystals formed in pores of 12% OPC mortar immersed at 25 °C in 10% Na_2_SO_4_ solution.

**Figure 16:**
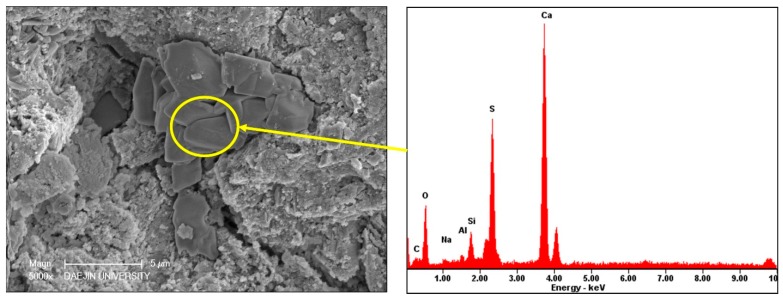
SEM image and EDS analyses of gypsum in 12% OPC mortar immersed at 25 °C in 10% Na_2_SO_4_ solution.

SEM investigations were conducted at the low and high resolution on the deteriorated specimens to investigate the internal microstructure deterioration. In the SEM image shown in Figure 14, aggregate and cement paste can be seen. The gaps around aggregates are cracks and expansion in the interfacial transition zone between the aggregate and cement paste. The cracks around the aggregates suggest that a sulfate attack caused expansion in the interfacial transition zone and damaged the bond between the aggregate and the paste. Cracks around aggregates in the outer zone are more prominent than cracks around aggregates in the inner zone. This suggests that the deterioration in samples is concentric. As samples were cylindrical, the peripheral zone was attacked first; expansive forces were then generated near the surface, causing cracks around aggregates and in the cement paste. These microcracks permitted the penetration of more sulfate ions into the microstructure. As incoming sulfate ions reacted with interior hydration products and other susceptible compounds, new crystals of ettringite and gypsum were formed in the air voids and in the cracks. As a result, the peripheral zone exhibited greater deterioration than the core. The gaps were likely filled with ettringite and gypsum. Figure 15 and Figure 16 show needle-like crystals of ettringite formed in the pores of cement paste and plate-like crystals of gypsum. EDS analysis conducted on the ettringite and gypsum crystals is also shown in the Figure 15 and Figure 16. A crack around the gypsum crystals is given in Figure 16. This crack is likely formed because of the crystallization pressure of gypsum. 

**Figure 17:**
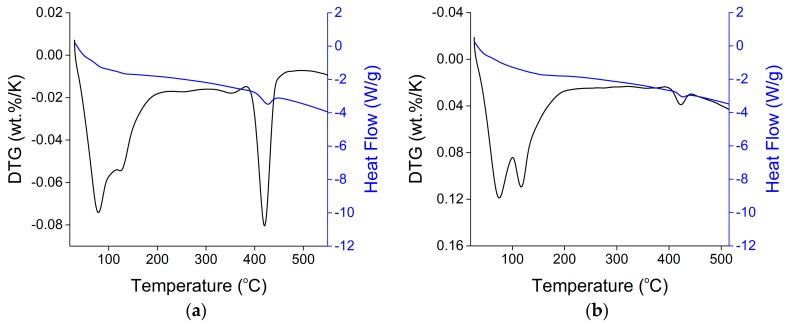
DTG and DSC curves in 12% OPC mortars stored in (**a**) saturated limewater; (**b**) 10% Na_2_SO_4_ solution.

Thermogravimetric analysis (TGA) and differential scanning calorimetry (DSC) analysis were conducted on 0%, 6%, 12% OPC and slag blended mortars stored in saturated limewater and sodium sulfate solution at 25 °C. Differential thermogravimetry (DTG) and differential scanning calorimetry (DSC) curves for 12% OPC mortars stored in saturated limewater and Na_2_SO_4_ solution at 25 °C for 84 days is shown in Figure 17. 

**Figure 18:**
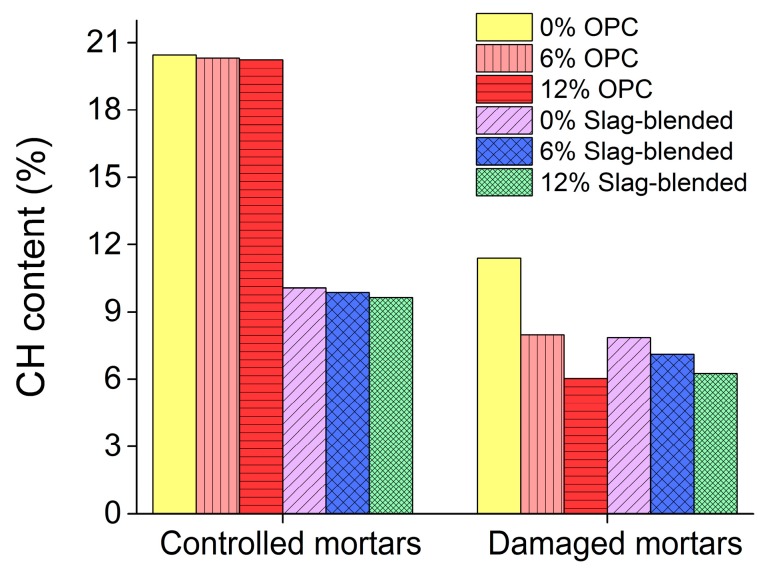
Portlandite content (%) in mortars stored in controlled and aggressive conditions.

**Figure 19:**
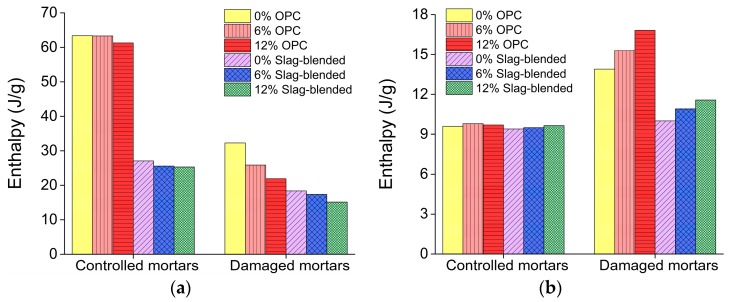
DSC results for (**a**) portlandite peaks at about 440 °C; (**b**) ettringite and gypsum peaks at 90–140 °C.

In Figure 17a, DTG curve shows two large peak peaks at 80 °C and 430 °C, these decompositions peaks are due to the dehydration of ettringite and portlandite respectively [28]. But after exposure to sulfate attack, the peak corresponding to the portlandite has been significantly reduced, a new peak has emerged at about 130 °C. This new peak is due to the decomposition of gypsum which was produced during sulfate attack. These new peaks can also be observed in XRD shown in Figure 12d. Figure 18 shows the percentage of portlandite in mortars stored in saturated limewater (controlled mortars) and Na_2_SO_4_ solution (damaged mortars) [28]. This figure shows that after sulfate attack, reduction in CH content is proportional to the increasing amount of nano-TiO_2_. Enthalpy of ettringite and gypsum corresponding to temperature 80–150 °C and portlandite corresponding to 400–500 °C was calculated from DSC curves [36] and is shown in Figure 19. A decreasing trend in enthalpy of CH in mortar samples after exposure to sulfate solution can be observed with the addition of nano-TiO_2_. Enthalpy of ettringite and gypsum are shown in Figure 19b. Contrary to CH enthalpy, here an increase in enthalpy can be observed with the addition of nano-TiO_2_ after sulfate attack. 

Crystallization pressure is considered to be responsible for the mechanism of expansion in sulfate attack [9]. When ettringite or gypsum crystals grow in a confined space, they exert an expansive force on the walls of the pores. According to Scherer [37], crystallization pressure is inversely proportional to the size of the pores. If the pores in which crystals grow are smaller in size, then the crystallization pressure will be higher. When the crystallization pressure is greater than the tensile strength of the mortar, the pores will expand and microcracks will occur. After microcracking, more sulfate ions will enter the sample, reacting with portlandite and forming gypsum.

TiO_2_ is an inert material [14,38], it does not react with water, cement and incoming sulfate ions. The nano-TiO_2_ particles act as nuclei for hydration reaction. The hydration products grow around them and fill the voids, thus reduce the porosity [14]. Mercury intrusion porosimetry (MIP) studies of nano-TiO_2_ containing cementitious materials [14,39,40,41,42] have shown that the addition of nano-TiO_2_ have refined their pores and the most probable pore diameters of cementitious materials have shifted towards smaller pore diameters. As stated above that during sulfate attack, expansive products (ettringite and gypsum) are created in the pores of the cement mortar and they create crystallization pressure on the walls of pores. If the size of pores is smaller, the crystallization pressure will be higher and vice versa. Here, the pore sizes were reduced due to the addition of nano-TiO_2_, so the crystallization pressure was higher due to the formation of ettringite and gypsum crystals in pores of nano-TiO_2_ containing mortars than control mortars. Secondly, the addition of nano-TiO_2_ particles can reduce the tensile strength of mortar [15]; therefore, the addition of nano-TiO_2_ not only increased crystallization pressure, which is a tensile force but also simultaneously reduced tensile strength, leaving mortars with nano-TiO_2_ more vulnerable to sulfate attack and salt crystallization pressure than normal mortars. According to Santhanam et al. [43], the presence of voids can reduce expansive stresses due to crystallization, which can reduce the number of cracks in the paste. The researchers [43] observed a slower rate of disintegration in the air-entrained mortars than in the non-air-entrained mortars. Therefore, nano-TiO_2_ containing mortars had less voids and more expansive stresses created due to crystallization pressure in smaller size voids and were ultimately more damaged than mortars without nano-TiO_2_. Lee and Kurtis [44] also concluded that the addition of nanoparticles increases the salt crystallization pressure. In their study, mortars containing nanoparticles showed greater damage due to salt crystallization than mortars without nanoparticles. TiO_2_ containing slag-blended mortars were resistant to sulfate attack because slag reacts with portlandite to form calcium silicate hydrate [31]. It consumes portlandite and increases the amount of CSH in mortars. Additional hydrates are formed in larger capillary pores and reduce pore connectivity [45]. This process likely reduced the porosity and permeability of TiO_2_ containing slag-blended mortars and increased the compressive and tensile strength of mortars [6]. The lower content of portlandite in slag-blended mortars can be observed in the XRD pattern shown in Figure 13. The other reactive hydration products susceptible to sulfate attack are monosulfoaluminate hydrate, calcium aluminate hydrate and calcium aluminate hydroxide. These products were likely in lower quantities in slag mortars due to the absence of C_3_A clinker phases in slag and these products are less reactive when present in slag [36]. As these phases are required for the formation of ettringite, their lower quantities in slag are not sufficient to cause ettringite-related expansion and cracking. The higher tensile strength of slag mortars (compared to OPC mortars) can also resist the expansion due to the formation of ettringite so nano-TiO_2_ containing slag-blended mortars were more resistant to sulfate attack than so nano-TiO_2_ containing OPC mortars.

## 4. Conclusions

In this study, we investigated the influence of titanium dioxide (TiO_2_) nanoparticles on the sulfate attack upon ordinary Portland cement and slag-blended mortars. Nano-TiO_2_ containing mortars were immersed in 10% Na_2_SO_4_ solution at 25 and 5 °C. The expansion, variation in mass and surface microhardness were observed. Deteriorated samples were analyzed using XRD, SEM, EDS, TGA and DSC tests. Based on the test results, the following points were concluded. 

Deterioration due to sulfate attack occurred on all TiO_2_ containing mortars. The results show that the addition of TiO_2_ has robust effects on the rate of sulfate attack on OPC and slag-blended mortars. The extent of damage increases with the addition of TiO_2_ nanoparticles. It is recommended that mortars and concrete structures containing TiO_2_ for self-cleaning and air purifying purposes should be designed as sulfate resistant.

The TiO_2_ containing slag-blended mortars showed lower expansion, mass and surface hardness changes and cracking. So, blast furnace slag can be used to reduce sulfate attack in nano-TiO_2_ containing mortars. 

The TiO_2_ containing mortar and concrete structures exposed to higher temperatures are more vulnerable to sulfate attack as the rate of expansion, mass variation and cracks were higher in mortars stored at 25 °C temperature than at 5 °C. Specimens at lower temperatures take more time to match the expansion of specimens at higher temperatures. 

The rate and severity of sulfate attack on TiO_2_ containing cementitious materials should be investigated by varying the factors like the type of cations of sulfate solution (K^+^, Mg^2+^, Ca^2+^), pH of the solution and size of the specimens. Also, the influence of titanium dioxide on sulfate attack upon other materials such as geopolymer concrete should be investigated.

## Figures and Tables

**Table 1:** Chemical composition and physical properties of ordinary Portland cement (OPC) and ground granulated blast furnace slag (GGBFS).

Oxide	OPC	GGBFS
CaO (%)	62.27	40.72
SiO_2_ (%)	21.32	34.86
Al_2_O_3_ (%)	5.19	12.54
SO_3_ (%)	2.17	1.32
MgO (%)	3.04	7.61
Fe_2_O_3_ (%)	2.23	0.72
K_2_O (%)	0.58	0.67
Na_2_O (%)	0.52	0.38
Loss on ignition	1.5	0.43
Blaine fineness (cm^2^/g)	3400	4600
Specific Gravity	3.15	2.9

**Table 2:** Mix proportion.

Acronym	OPC (g)	Slag (g)	TiO_2_ (g)	Sand (g)	Water/Binder Ratio	Water Reducing Admixture (%)
0% OPC	1500	0	0	4500	0.40	0
3% OPC	1500	0	45	4500	0.40	0.25
6% OPC	1500	0	90	4500	0.40	0.6
9% OPC	1500	0	135	4500	0.40	1
12% OPC	1500	0	180	4500	0.40	1.2
0% slag-blended	750	750	0	4500	0.40	0
3% slag-blended	750	750	45	4500	0.40	0.2
6% slag-blended	750	750	90	4500	0.40	0.55
9% slag-blended	750	750	135	4500	0.40	1
12% slag-blended	750	750	180	4500	0.40	1.2

**Table 3:** Damage rating in nano-TiO_2_ containing OPC and slag-blended mortar specimens stored at 25 and 5 °C in Na_2_SO_4_ solution.

Name	25 °C				5 °C			
	0 Day	28 Days	56 Days	84 Days	0 Day	28 Days	56 Days	84 Days
0% OPC	0	1	3	4	0	1	2	3
3% OPC	0	1	3	4	0	1	2	4
6% OPC	0	1	3	4	0	1	3	4
9% OPC	0	2	3	5	0	1	3	4
12% OPC	0	2	4	6	0	2	3	5
0% slag-blended	0	0	1	2	0	0	1	2
3% slag-blended	0	0	1	2	0	0	1	2
6% slag-blended	0	0	1	2	0	0	1	2
9% slag-blended	0	0	2	3	0	0	1	2
12% slag-blended	0	0	2	3	0	0	1	2

0: No crack visible; 1: Slight cracks; 2: Some cracks; 3: Moderate cracks; 4: Severe cracks and some map cracks; 5: Severe cracks and moderate map cracks; 6: Severe cracks and minor spalling; 7: Intensive cracks and severe spalling.

## References

[1] Chen J., Poon C.-S. (2009). Photocatalytic construction and building materials: From fundamentals to applications. Build. Environ..

[2] Chen J., Kou S.-C., Poon C.-S. (2011). Photocatalytic cement-based materials: Comparison of nitrogen oxides and toluene removal potentials and evaluation of self-cleaning performance. Build. Environ..

[3] Strini A., Roviello G., Ricciotti L., Ferone C., Messina F., Schiavi L., Corsaro D., Cioffi R. (2016). TiO_2_-based photocatalytic geopolymers for nitric oxide degradation. Materials.

[4] Ohama Y., Van Gemert D. (2011). Application of Titanium Dioxide Photocatalysis to Construction Materials: State-of-the-Art Report of the Rilem Technical Committee 194-TDP.

[5] Hüsken G., Hunger M., Brouwers H. (2009). Experimental study of photocatalytic concrete products for air purification. Build. Environ..

[6] Siddique R., Khan M.I. (2011). Supplementary Cementing Materials.

[7] Virgalitte S.J., Luther M.D., Rose J.H., Mather B., Bell L.W., Ehmke B.A., Klieger P., Roy D.M., Call B.M., Hooton R.D. (1995). Ground Granulated Blast-Furnace Slag as a Cementitious Constituent in Concrete.

[8] Bastos G., Patiño-Barbeito F., Patiño-Cambeiro F., Armesto J. (2016). Nano-inclusions applied in cement-matrix composites: A review. Materials.

[9] Yu C., Sun W., Scrivener K. (2013). Mechanism of expansion of mortars immersed in sodium sulfate solutions. Cem. Concr. Res..

[10] Gruyaert E., Van den Heede P., Maes M., De Belie N. (2012). Investigation of the influence of blast-furnace slag on the resistance of concrete against organic acid or sulphate attack by means of accelerated degradation tests. Cem. Concr. Res..

[11] Santhanam M., Cohen M.D., Olek J. (2001). Sulfate attack research—Whither now?. Cem. Concr. Res..

[12] Neville A. (2004). The confused world of sulfate attack on concrete. Cem. Concr. Res..

[13] Marchand J., Odler I., Skalny J.P. (2003). Sulfate Attack on Concrete.

[14] Chen J., Kou S.-C., Poon C.-S. (2012). Hydration and properties of nano-TiO_2_ blended cement composites. Cem. Concr. Compos..

[15] Lucas S., Ferreira V., de Aguiar J.B. (2013). Incorporation of titanium dioxide nanoparticles in mortars—Influence of microstructure in the hardened state properties and photocatalytic activity. Cem. Concr. Res..

[16] Meng T., Yu Y., Qian X., Zhan S., Qian K. (2012). Effect of nano-TiO_2_ on the mechanical properties of cement mortar. Constr. Buil. Mater..

[17] Lee B.Y. (2012). Effect of Titanium Dioxide Nanoparticles on Early Age and Long Term Properties of Cementitious Materials.

[18] Pérez-Nicolás M., Balbuena J., Cruz-Yusta M., Sánchez L., Navarro-Blasco I., Fernández J.M., Alvarez J.I. (2015). Photocatalytic NO_X_ abatement by calcium aluminate cements modified with TiO_2_: Improved NO_2_ conversion. Cem. Concr. Res..

[19] (2018). Standard Test Method for Length Change of Hydraulic-Cement Mortars Exposed to a Sulfate Solution American.

[20] Van Tittelboom K., De Belie N., Hooton R.D. (2013). Test methods for resistance of concrete to sulfate attack—A critical review. Performance of Cement-Based Materials in Aggressive Aqueous Environments.

[21] Ferraris C., Stutzman P., Peltz M., Winpigler J. (2005). Developing a more rapid test to assess sulfate resistance of hydraulic cements. J. Res. Natl. Inst. Stand. Technol..

[22] Ferraris C.F., Clifton J.R., Stutzman P.E., Garboczi E. (1997). Mechanisms of Chemical Degradation of Cement-Based Systems.

[23] (2016). Standard Test Method for Compressive Strength of Hydraulic Cement Mortars (Using 2-in. or [50-mm] Cube Specimens).

[24] (2002). Standard Test Method for Flexural Strength and Modulus of Hydraulic Cement Mortars.

[25] (2013). Standard Test Method for Density, Absorption and Voids in Hardened Concrete.

[26] Igarashi S., Bentur A., Mindess S. (1996). Microhardness testing of cementitious materials. Adv. Cem. Based Mater..

[27] Sarkar S.L., Aimin X., Jana D. (2001). Scanning electron microscopy, X-ray microanalysis of concretes-7. Handbook of Adhesives Raw Materials.

[28] Lothenbach B., Durdzinski P., De Weerdt K. (2016). Thermogravimetric analysis. A Practical Guide to Microstructural Analysis of Cementitious Materials.

[29] Nazari A., Riahi S. (2010). The effect of TiO_2_ nanoparticles on water permeability and thermal and mechanical properties of high strength self-compacting concrete. Mater. Sci. Eng. A.

[30] Hooton R.D. (2000). Canadian use of ground granulated blast-furnace slag as a supplementary cementing material for enhanced performance of concrete. Can. J. Civ. Eng..

[31] Mehta P.K. (1986). Concrete: Structure, Properties and Materials.

[32] Bouikni A., Swamy R.N., Bali A. (2009). Durability properties of concrete containing 50% and 65% slag. Constr. Build. Mater..

[33] Lang E., Bensted J., Barnes P. (2002). Blast furnace cements. Structure and Performance of Cements.

[34] Menéndez E., Matschei T., Glasser F.P. (2013). Sulfate attack of concrete. Performance of Cement-Based Materials in Aggressive Aqueous Environments.

[35] Whittaker M., Zajac M., Ben Haha M., Black L. (2016). The impact of alumina availability on sulfate resistance of slag composite cements. Constr. Build. Mater..

[36] Bassuoni M.T., Nehdi M.L. (2009). Durability of self-consolidating concrete to sulfate attack under combined cyclic environments and flexural loading. Cem. Concr. Res..

[37] Scherer G.W. (1999). Crystallization in pores. Cem. Concr. Res..

[38] Jayapalan A., Lee B., Fredrich S., Kurtis K. (2010). Influence of additions of anatase TiO_2_ nanoparticles on early-age properties of cement-based materials. Transp. Res. Rec. J. Transp. Res. Board.

[39] Yang L.Y., Jia Z.J., Zhang Y.M., Dai J.G. (2015). Effects of nano-TiO_2_ on strength, shrinkage and microstructure of alkali activated slag pastes. Cem. Concr. Compos..

[40] Zhang R., Cheng X., Hou P., Ye Z. (2015). Influences of nano-TiO_2_ on the properties of cement-based materials: Hydration and drying shrinkage. Constr. Build. Mater..

[41] Jimenez-Relinque E., Rodriguez-Garcia J.R., Castillo A., Castellote M. (2015). Characteristics and efficiency of photocatalytic cementitious materials: Type of binder, roughness and microstructure. Cem. Concr. Res..

[42] Nazari A., Riahi S. (2011). TiO_2_ nanoparticles effects on physical, thermal and mechanical properties of self compacting concrete with ground granulated blast furnace slag as binder. Energy Build..

[43] Santhanam M., Cohen M.D., Olek J. (2003). Mechanism of sulfate attack: A fresh look: Part 2. Proposed mechanisms. Cem. Concr. Res..

[44] Lee B.Y., Kurtis K.E. (2017). Effect of pore structure on salt crystallization damage of cement-based materials: Consideration of w/b and nanoparticle use. Cem. Concr. Res..

[45] Divsholi B.S., Lim T.Y.D., Teng S. (2014). Durability properties and microstructure of ground granulated blast furnace slag cement concrete. Int. Concr. Struct. Mater..

